# A Preprocessing Manifold Learning Strategy Based on t-Distributed Stochastic Neighbor Embedding

**DOI:** 10.3390/e25071065

**Published:** 2023-07-14

**Authors:** Sha Shi, Yefei Xu, Xiaoyang Xu, Xiaofan Mo, Jun Ding

**Affiliations:** 1 State Key Laboratory of Integrated Services Network, Xidian University, 2 South TaiBai Road, Xi’an 710071, China; sshi@xidian.edu.cn (S.S.); xyxu510@stu.xidian.edu.cn (X.X.); 2 National Astronomical Observatories, Chinese Academy of Sciences, 20A Datun Road, Chaoyang District, Beijing 100101, China; moxiaofan@nao.cas.cn; 3 Institute of Information Sensing, Xidian University, 2 South TaiBai Road, Xi’an 710071, China

**Keywords:** manifold learning, t-SNE, dimensionality reducing, k-nearest neighbor

## Abstract

In machine learning and data analysis, dimensionality reduction and high-dimensional data visualization can be accomplished by manifold learning using a t-Distributed Stochastic Neighbor Embedding (t-SNE) algorithm. We significantly improve this manifold learning scheme by introducing a preprocessing strategy for the t-SNE algorithm. In our preprocessing, we exploit Laplacian eigenmaps to reduce the high-dimensional data first, which can aggregate each data cluster and reduce the Kullback–Leibler divergence (KLD) remarkably. Moreover, the k-nearest-neighbor (KNN) algorithm is also involved in our preprocessing to enhance the visualization performance and reduce the computation and space complexity. We compare the performance of our strategy with that of the standard t-SNE on the MNIST dataset. The experiment results show that our strategy exhibits a stronger ability to separate different clusters as well as keep data of the same kind much closer to each other. Moreover, the KLD can be reduced by about 30% at the cost of increasing the complexity in terms of runtime by only 1–2%.

## 1. Introduction

In machine learning and other computer-related areas, the demands for dimensionality reduction methods never vanish owing to the curse of dimensionality [1,2,3]. Generally, the amount of calculation often grows exponentially with the increase in dimensionality, hence the efficiency of machine learning algorithms will drop markedly if the dimension of the input data is enormous [2,4]. On account of the limited computing power at present, it is essential to devise dimensionality reduction methods to obtain a sound and reliable result. On the other hand, in many realms, it is also of great interest to reduce high-dimensional data to two or three dimensions for visualization purposes [5,6,7,8,9].

For decades, a large number of dimensionality reduction methods have been applied to different tasks, among them are Principal Component Analysis (PCA) [10,11,12], Multidimensional Scaling (MDS) [13,14], Sammon Mapping [15], Isomap [16], Locally Linear Embedding (LLE) [17], Laplacian Eigenmaps (LE) [18,19,20], t-Distributed Stochastic Neighbor Embedding (t-SNE) [21,22,23,24] and so on. It is well known that the first three algorithms mentioned above are linear dimensionality reduction methods, which usually break the inner data structure of real-world datasets, thus yielding a poor visualization map. The others are non-linear and can be concluded to be manifold learning algorithms.

Manifold learning tends to outperform linear dimensionality reduction methods in data visualization. Particularly, t-SNE is amongst the best-known manifold learning algorithms, as it can not only capture much of the local structure of the high-dimensional data but also reveal the associated global structure, such as by presenting clusters at many different scales [21].

As unprecedented as the performance of t-SNE is, however, several problems still remain to be addressed. Firstly, owing to the assumption in t-SNE that the high-dimensional data are in Gaussian distribution, the distribution of the mapped data in low dimensions is always uniform and loose, and the Kullback–Leibler divergence (KLD) often converges to a high value, which prevents the algorithm from generating a sound low-dimensional map. Secondly, separations between different natural clusters still need to be improved as some of the data are often inclined to be clustered into the wrong groups due to the obscure boundary. Thirdly, both the computation and space complexity of t-SNE increase quadratically with the number of data pairs, which severely limits the application of t-SNE on large datasets in reality.

In this paper, we significantly improve the standard t-SNE scheme by developing a preprocessing strategy for it. In our preprocessing strategy, Laplacian eigenmaps (LE) are first employed on the high-dimensional data. Thus, before they are input into t-SNE, each data cluster can be aggregated first and the data are no longer in Gaussian distribution.

In addition, aiming at magnifying the gaps between different clusters and enlarging the difference between data of different kinds, the K-nearest-neighbor (KNN) algorithm is also introduced into our preprocessing to shrink the Euclidean distance between each neighboring data pair. Moreover, compared to the standard t-SNE, KNN is also expected to reduce the computation and space complexity as only the neighboring data pairs are considered in our strategy, which can offer a balance between performance and complexity.

We apply our method on the MNIST dataset, which contains 70,000 handwritten numeric images that are 28 pixels by 28 pixels in size. The training set contains 60,000 images, while the test set 10,000 images. The experimental results show that our strategy can significantly improve the performance of the standard t-SNE and the recoveries of low-dimensional data structures are also reinforced, while the overall complexity only increases by about 1–2%.

The outline of this paper is as follows: Section 2 gives a quick review of the basic idea of the standard t-SNE. In Section 3, a preprocessing manifold learning strategy based on t-SNE, LE and KNN is proposed. The numerical results on the MNIST dataset are presented in Section 4. Finally, we draw some conclusions in Section 5.

## 2. Manifold Learning by Using a t-SNE

Generally speaking, dimensionality reduction methods convert the high-dimensional dataset $X=\{x_{1},x_{2},\cdots ,x_{n}\}$ into two- or three-dimensional data $Y=\{y_{1},y_{2},\cdots ,y_{n}\}$ that can be displayed in a scatterplot. It is argued in [25] that a set of similar data is neither randomly nor uniformly distributed in this space, but instead lies on or near a submanifold of much lower dimension. Manifold learning is a sort of non-linear technique that aims to find a non-linear mapping to extract the intrinsic dimensionality of the original data and to realize dimensionality reduction.

For traditional dimensionality reduction methods, such as the Locally Linear Embedding algorithm, the similarity between data is typically modeled by Euclidean distance. Thus, it is difficult for those traditional dimensionality reduction methods to unfold “many-to-one” mappings, in which a single ambiguous object really belongs in several disparate locations in the low-dimensional space [26]. To solve the problem, the t-SNE algorithm proposed by Maaten and Hinton  [21] employs a probabilistic model to visualize the structure of complex datasets. Specifically, t-SNE converts high-dimensional Euclidean distances between data points into joint probabilities to characterize the similarities between data.

In t-SNE, the conditional probabilities $p_{j|i}$ that a data point $x_{i}$ would pick $x_{j}$ as its neighbor is given by [27]

(1) $$p_{j|i}=\frac{exp(-||x_{i}-x_{j}|{|}^{2}/2\sigma_{i}^{2})}{\sum_{j\neq i}exp(-||x_{i}-x_{j}{\left|\right|}^{2}/2\sigma_{i}^{2})}$$

with $p_{i|i}=0$. The variance $\sigma_{i}$ in the Gaussians centered around $x_{i}$ is determined by a binary search procedure. The data density is likely to vary. Thus, in dense regions, a smaller value of σ is more appropriate than that in sparse regions.

Let $P_{i}$ be the conditional probability distribution over all other data points at a given point $x_{i}$. The entropy of $P_{i}$ will grow along with the increase in $\sigma_{i}$. Then, with a fixed perplexity specified by the user, a binary search for $\sigma_{i}$ is performed in t-SNE to produce a probability distribution $P_{i}$. In a sense, the perplexity can be interpreted as a smooth measure of the effective number of neighbors. The performance of t-SNE is fairly robust to changes in the perplexity, which is typically between 5 and 50, depending on the size of the datasets. The joint probabilities $p_{ij}$ can be obtained easily as follows:

(2) $$p_{ij}=\frac{p_{j|i}+p_{i|j}}{2},$$

which ensures that $\sum_{j}p_{ij}>\frac{1}{2n}$ for all data points $x_{i}$. Thus, each data point $x_{i}$ can make a significant contribution to the cost function [21].

In the lower dimension, t-SNE employs a Student t-distribution with one degree of freedom as the heavy-tailed distribution to separate different clusters from each other. The joint probabilities of map points are given by [27]

(3) $$q_{j|i}=\frac{\left(1+|\right|y_{i}-y_{j}{\left|\right|}^{2}{)}^{-1}}{\sum_{k\neq l}exp(1+||y_{k}-y_{l}{\left|\right|}^{2}{)}^{-1}}.$$

KLD is typically adopted to characterize the mismatch between $p_{ij}$ and $q_{j|i}$. t-SNE minimizes the sum of KLD over all data points using a gradient descent method. The cost function *C* and the gradient of t-SNE are given by [27]

(4) $$C=KL\left(P||Q\right)=\sum_{i}\sum_{j}p_{ij}log\frac{p_{ij}}{q_{ij}},$$

and

(5) $$\frac{\delta C}{\delta y_{i}}=4\sum_{j}\left(p_{ij}-q_{ij}\right)\left(y_{i}-y_{j}\right)\left(1+|\right|y_{i}-y_{j}{\left|\right|}^{2}{)}^{-1},$$

respectively. Once the KLD decreases to an appropriate value, a faithful low-dimensional map will be obtained.

## 3. The Proposed Preprocessing Manifold Learning Strategy

We noticed that the drawbacks of the t-SNE mentioned above are partly caused by the Gaussian distribution of the high-dimensional data. In other words, due to the algorithm design of t-SNE, the mapped data will be uniformly but loosely distributed in a low dimension given that the high-dimensional data are in Gaussian distribution. Thus, a natural way to handle such a problem is to modify the original data distribution in advance in a reasonable way to fit the standard t-SNE. By reasonable, we mean the updated data distribution tailored to t-SNE also inherits the characteristics of the original data distribution.

Laplacian eigenmaps is another efficient manifold learning method, which maps the high-dimensional data into a lower dimension by solving a generalized eigenvector problem [19]. It shares some similar properties with LLE, e.g., it also employs weights rather than the possibility to realize dimensionality reduction, hence a tighter map compared with t-SNE could be obtained. Thus, the idea of LE can be introduced here to preprocess the original high-dimensional data. Following the dimensionality reduction strategy of LE [28], we first find the k-nearest neighbors for each data point by using KNN and characterize their relations with $W_{ij}$ as follows:

$$W_{ij}=\left\{\begin{array}{cc} e^{-\frac{\left|\right|x_{i}-x_{j}{\left|\right|}^{2}}{t}} & \mathrm{if}i,j\mathrm{are}\mathrm{neighbors},\\ 0 & \mathrm{otherwise}. \end{array}\right.$$

Then the cost function is given by

(6) $$\sum_{i=1}^{N}\sum_{j=1}^{N}\left|\right|y_{i}-y_{j}{\left|\right|}^{2}W_{ij}.$$

To minimize the cost function, there are three matrices D, W and L defined in LE, in which D is a diagonal matrix where $D_{ii}=\sum_{j}W_{ij}$. W is composed of $W_{ij}$, where $W_{ij}$ is just the element at row *i* and column *j*. The Laplacian matrix [28] is defined as

(7) L=D−W.

Thus, the first dimensionality reduction can be accomplished by solving a generalized eigenvector problem, i.e.,

(8) Ly=λDy,

and the m-dimensional mapped data are corresponding to the smallest *m* non-zero eigenvalues in Equation (8) [28].

It is easy to see that by using LE the data points can be aggregated more tightly with their neighbors and the mapped data are not in Gaussian distribution anymore. Thus, if we take the mapped data that were preprocessed by LE as the input of t-SNE, the loose distribution problem associated with t-SNE can be significantly alleviated.

**Figure 1:**
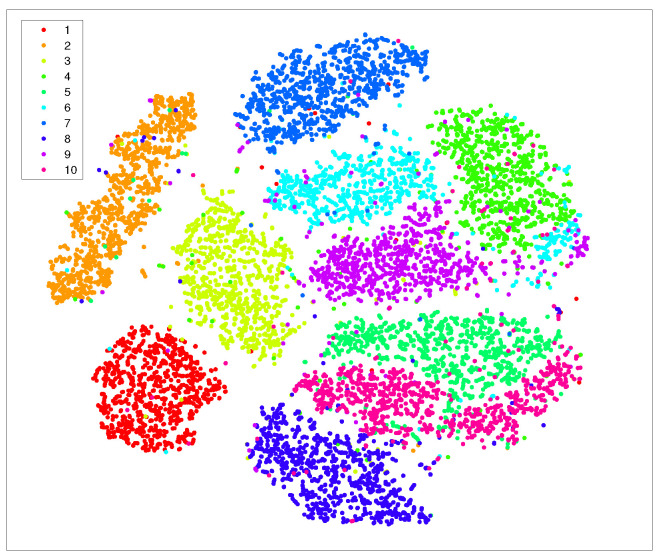
Visualization of 5000 data randomly selected from MNIST by using t-SNE, the iteration number is 500.

**Figure 2:**
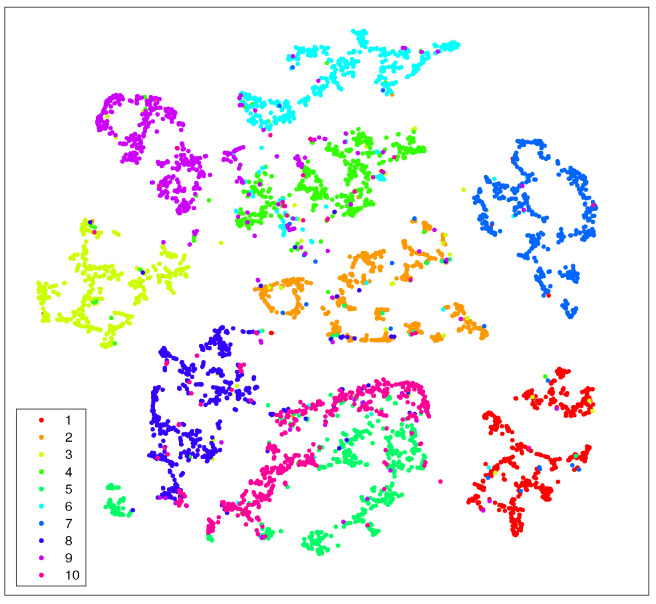
Visualization of 5000 data randomly selected from MNIST by using t-SNE with LE as the preprocessing strategy. Here we take $k_{0}=40$ and $N_{0}=80$. The iteration number of t-SNE is also 500.

In Figure 1 and Figure 2, we show the visualization of 5000 data selected from the MNIST randomly by using t-SNE and t-SNE with LE as the preprocessing strategy, respectively. The procedure of the latter strategy is as follows: We first use KNN to obtain $k_{0}$ neighbors for each data point; then, we reduce the dimensionality of the original data to $N_{0}$ using the concept of LE. Finally, we take those $N_{0}$-dimensional data as the input for t-SNE to further accomplish the dimensionality reduction.

Just as we expect, the t-SNE implements t-distribution to solve the crowd problem and a relatively obvious gap between different data clusters can be formed. However, the clusters themselves are still loose to some extent, which undermines the ability of t-SNE to form tight clusters. Worse yet, the entropy of each cluster is too high to yield a small K–L divergence. On the other hand, from Figure 2, it is evident that not only the gaps between each of the ten data clusters can be formed by using the preprocessing strategy, but each data point also tends to be gathered with its neighboring points.

However, we notice that the gaps between different clusters are not satisfactory, both for the standard t-SNE and the one with LE as the preprocessing strategy, as shown in Figure 1 and Figure 2. Moreover, as both the computation and space complexity of t-SNE grow quadratically with the number of data pairs, it will be of great interest to reduce the number of data pairs involved in the final t-SNE.

Here, we continue to preprocess the data that experienced dimensionality reduction by LE before they are processed by t-SNE. Inspired by the sparse approximation strategy proposed in [29], we again use the KNN algorithm to find out the neighbors for each data point, then aggregate neighboring similar data points and weaken the relationships between dissimilar data pairs. As in [29], the pairwise similarities between data points, $p_{ij}$, is redefined by

(9) $$p_{j|i}=\left\{\begin{array}{cc} \frac{exp(-{\left||x_{i}-x_{j}|\right|}^{2}/2\sigma_{i}^{2})}{\sum_{j\neq i}exp(-||x_{i}-x_{j}{\left|\right|}^{2}/2\sigma_{i}^{2})} & \mathrm{if}i,j\mathrm{are}\mathrm{neighbors}\\ 0 & \mathrm{otherwise} \end{array}\right.$$

In this way, we propose another strategy by which we can decrease the distance between neighboring data in order to increase the possibility that a data point chooses its real neighbor.

After we implement LE, we perform the KNN algorithm again to find the neighbors of each data point, and then we introduce a coefficient α as below

(10) $$d_{ij}=\left\{\begin{array}{cc} \alpha ||x_{i}-x_{j}{\left|\right|}^{2} & \mathrm{if}i,j\mathrm{are}\mathrm{neighbors}\\ \frac{1}{\alpha }\left|\right|x_{i}-x_{j}{\left|\right|}^{2} & \mathrm{otherwise} \end{array}\right.$$

It makes sense and does little harm to the original data structure since we have already implemented Laplacian eigenmaps to preprocess the data. With regard to the MNIST dataset, we set α as $1\times 10^{-5}$.

Now, we present our dimensionality reduction strategy with preprocessing as shown in Algorithm 1.
**Algorithm** **1** A preprocessing manifold learning strategy based on t-SNE.**Input:** The high-dimensional data set X.**Output:** The two-dimensional data Y after the dimensionality reduction. 1:Compute the Euclidean distances for each of the high-dimensional data pairs; 2:Apply a KNN algorithm to find out $k_{0}$ neighbors for each data; 3:Apply Laplacian Eigenmaps on the original data and reduce its dimensionality to $N_{0}$; 4:Apply the KNN algorithm again to find out $k_{1}$ neighbors for each data, and for two data points that are not neighbors i,j, set their conditional probability $p_{j|i}$ to 0; 5:Decrease the distance between neighboring data points by applying the coefficient α on the neighboring data points; 6:Compute the joint probabilities with $p_{ij}=\frac{p_{j|i}+p_{i|j}}{2}$; 7:Get the low-dimensional mapped data $Y^{\left(0\right)}$; 8:Compute the joint probabilities of the mapped points with $q_{j|i}=\frac{\left(1+|\right|y_{i}-y_{j}{\left|\right|}^{2}{)}^{-1}}{\sum_{k\neq l}exp(1+||y_{k}-y_{l}{\left|\right|}^{2}{)}^{-1}}$; 9:Compute $\frac{\delta C}{\delta y_{i}}=4\sum_{j}\left(p_{ij}-q_{ij}\right)\left(y_{i}-y_{j}\right)\left(1+|\right|y_{i}-y_{j}{\left|\right|}^{2}{)}^{-1}$;10:Compute $Y^{\left(t\right)}=Y^{\left(t-1\right)}+\eta \frac{\delta C}{\delta Y}+\alpha \left(t\right)\left(Y^{\left(t-1\right)}-Y^{\left(t-2\right)}\right)$, where η represents the learning rate and α(t) the momentum;11:Repeat steps 8, 9 and 10 until *t* reach the number of iterations to implement t-SNE on the pre-processed data or the corresponding dimensionality of the mapped data is reduced to be 2.

**Figure 3:**
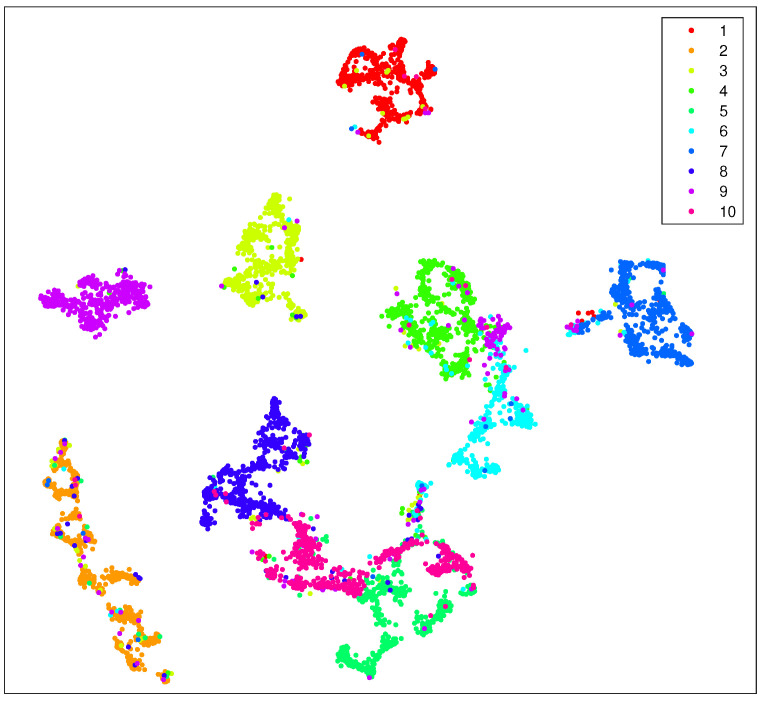
Visualization of 5000 data selected from MNIST randomly with our preprocessing strategy as shown in Algorithm 1. Here, we take $k_{0}=k_{1}=40$, $N_{0}=80$ and the iteration number is 500. During the first 100 iteration of gradient descent, early exaggeration is exploited.

In Figure 3, we show the visualization of 5000 data points selected from MNIST randomly with our preprocessing strategy. The procedure of our preprocessing strategy is as follows: first, we use KNN to obtain $k_{0}$ neighbors for each data point; then, we apply LE to reduce the dimensionality of the original data; after that, we implement the KNN algorithm again to find $k_{1}$ neighbors for each data point; and finally, we apply t-SNE on the preprocessed data and reduce their dimensionality to 2.

**Figure 4:**
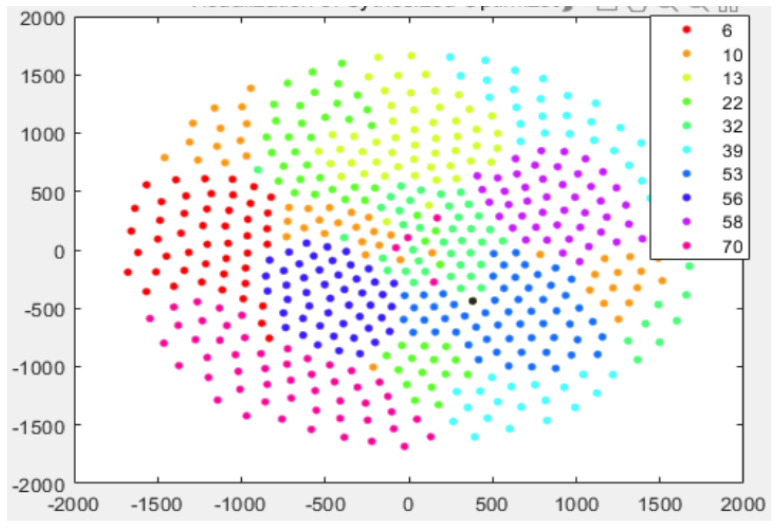
Visualization of 720 data from 10 objects randomly selected from Coil−100 by using t−SNE.

**Figure 5:**
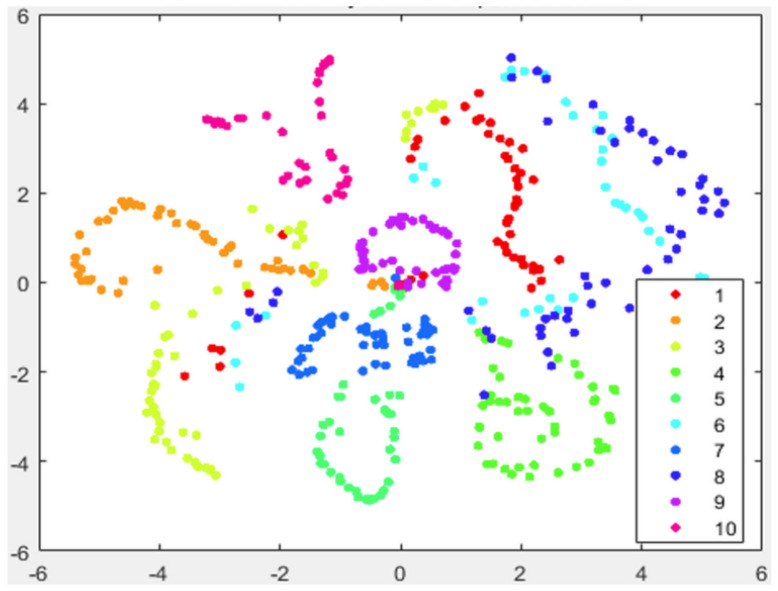
Visualization of 720 data from 10 objects randomly selected from Coil−100 with our preprocessing strategy.

**Figure 6:**
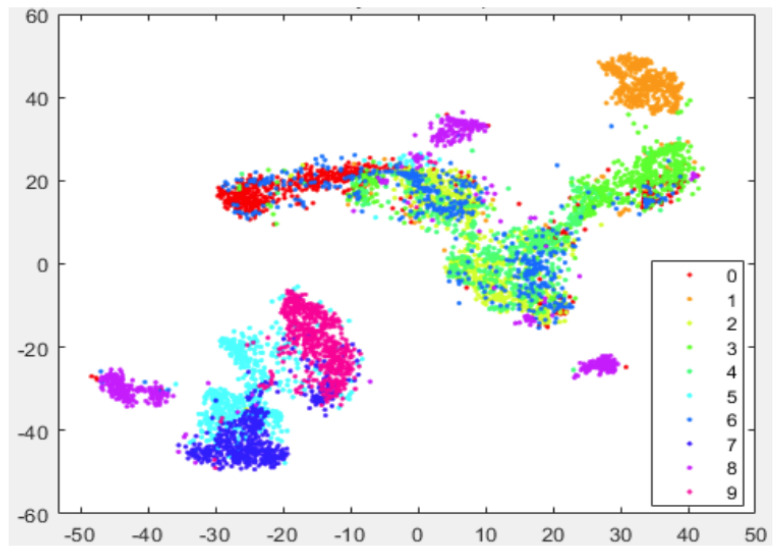
Visualization of 5000 data randomly selected from Fashion−MNIST by using t−SNE.

**Figure 7:**
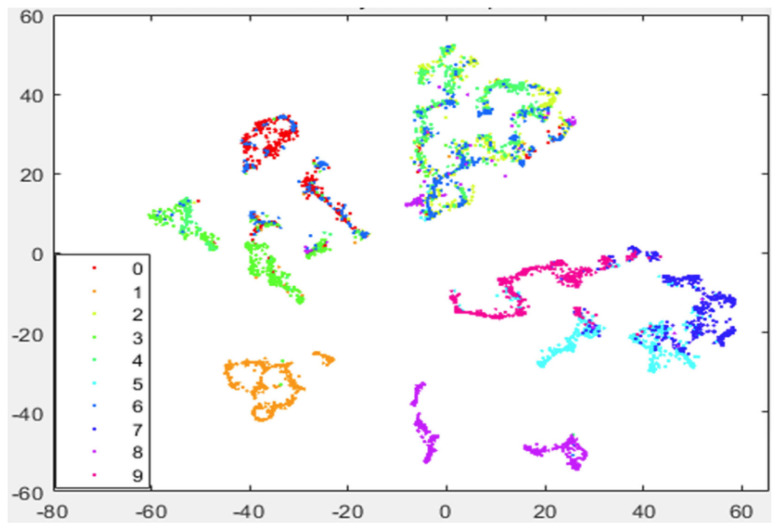
Visualization of 5000 data randomly selected from Fashion−MNIST with our preprocessing strategy.

We have also applied our strategies on datasets Coil-100 and the Fashion-MNIST. The former is a collection of color images of 100 objects taken from different angles and comprises 72 images per object in total. The size of each image is uniformly processed to be 128×128. The latter is a clothing image dataset from Zalando, Berlin, Germany, containing a training set of 60,000 samples and a test set of 10,000 samples. Each sample is a 28×28 grayscale image associated with 10 categories of labels. The performance of the t-SNE algorithm and our strategy on these datasets are shown in Figure 4, Figure 5, Figure 6 and Figure 7. According to the simulation results, our strategy has better performance on the MNIST dataset and the Fashion-MNIST dataset. For the Coil-100 dataset, though the performance of our strategy is slightly unsatisfactory due to the high dimensionality of the data, it is better than the standard t-SNE algorithm.

## 4. Discussion

As in previous simulations, in Figure 3 we again show the visualization of 5000 data selected from MNIST randomly but by using our approach (Algorithm 1). It can be seen that, compared with Figure 2, our strategy shows more pronounced benefits since data of the same kind are aggregated far more tightly. In addition, the gaps between different clusters are also enlarged. Thus, it provides many more advantages for the process of extracting each cluster respectively.

**Figure 8:**
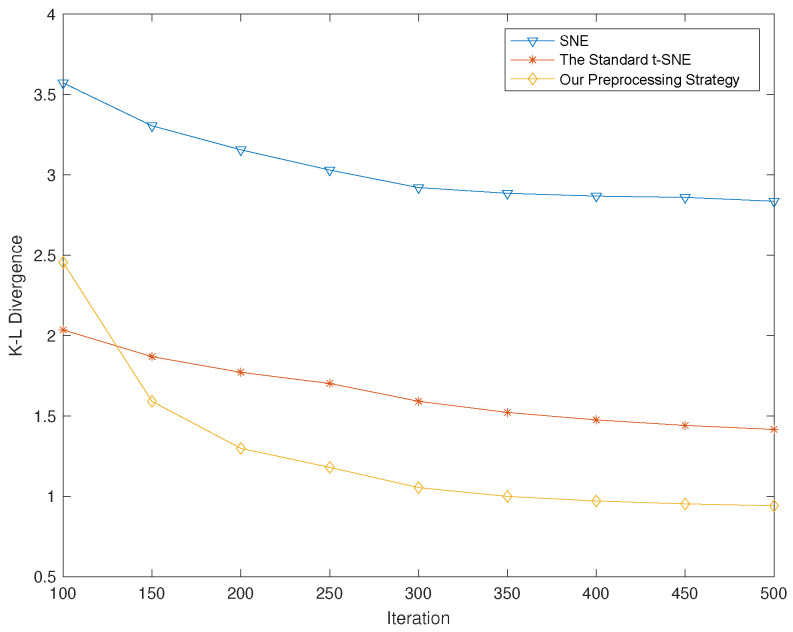
The gradient descent process of the SNE, the standard t-SNE and our preprocessing strategy.

Just as we mentioned in the last section, the strategy by which we decrease the distance between neighboring data destroys the data structure in some cases, leading to an increase in KLD to some extent. However, the KLD can be significantly reduced by using the idea of LE in step 3 in Algorithm 1. In Figure 8, we show the changing trend of KLD along with the increase in iterations. It is evident that by using our preprocessing strategy, the KLD plateaus around “1” after 500 iterations, while the standard t-SNE plateaus at around 1.5 and the SNE plateaus at 2.8. In other words, the KLD yielded by our approach is about 33% lower than the standard t-SNE and almost 66% lower than the SNE.

We also compare the computation complexity of the three approaches mentioned in the context in terms of running time. As shown in Table 1, the running time caused by our approach is increased by only 1.82% when compared with the standard t-SNE. There are two reasons for this. Firstly, LE is a highly efficient algorithm; when it is introduced into our preprocessing strategy to accomplish the preliminary dimensionality reduction, the extra complexity can almost be ignored. Secondly, though KNN algorithms are used in our approach twice, the number of data pairs considered in t-SNE can also be reduced correspondingly. In other words, the extra complexity caused by KNN can be offset by itself.

For the space complexity, since we have added a preprocessing process to the standard t-SNE algorithm, some extra space is required, which is mainly caused by the Laplacian eigenmaps and the KNN algorithm. Firstly, for the KNN algorithm, each data point is stored as a separate object or array, and its space complexity is O(n×d), where *n* represents the number of data points and *d* represents the dimensionality of each data point. Secondly, Laplacian eigenmaps need to construct the graph Laplacian matrix, which is an n×n matrix, so the space complexity of Laplacian eigenmaps is $O(n^{2})$. As the standard t-SNE needs to compute and store the joint probabilities of mapped points, its space complexity is $O(n^{2})$. In conclusion, our strategy requires approximately twice as much space compared to the standard t-SNE algorithm.

**Figure 9:**
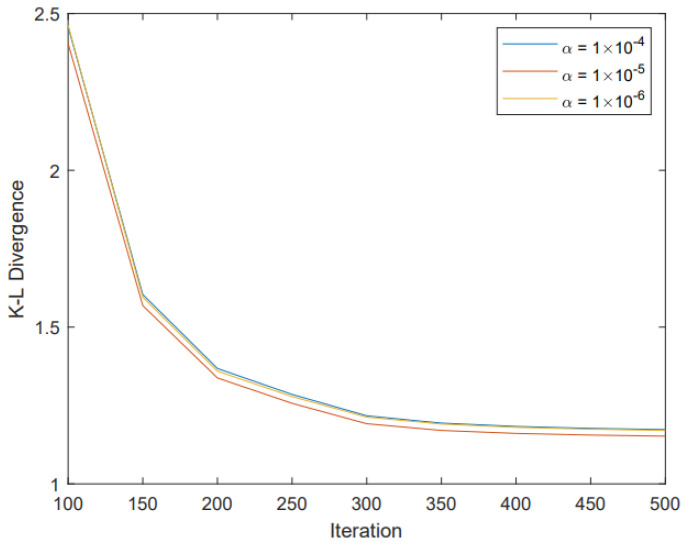
The gradient descent process of our preprocessing strategy when using different parameters α.

**Figure 10:**
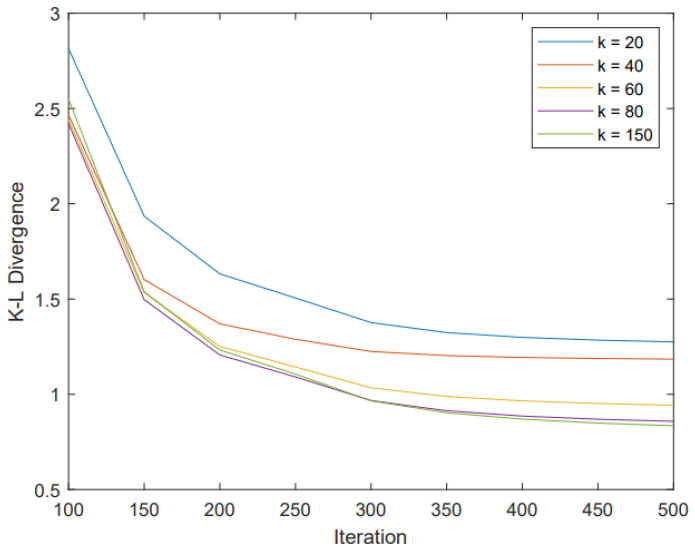
The gradient descent process of our preprocessing strategy when using different parameters k, where we let k = $k_{0}$ = $k_{1}$.

To show the effect of different parameters $k_{0}$, $k_{1}$ and α on the performance of our algorithm, we performed simulations with different parameters and gave the corresponding gradient descent process. Since both $k_{1}$ and $k_{2}$ are the neighbor numbers of the KNN algorithm, we also made them consistent to maintain their consistency. In Figure 9, we show that a suitable parameter α can bring good performance to our strategy. In Figure 10, we show that the performance does become better along with the increase in $k_{0}$ and $k_{1}$, but at a cost of higher complexity as more neighbor nodes are involved. On the other hand, we find that when $k_{0}$ and $k_{1}$ are increased to 80, the performance reaches a saturated state.

Moreover, as we can set those parameters ($k_{0}$, $k_{1}$ and $N_{0}$) according to different scenarios, our approach offers a flexible balance between complexity and performance as required. Now, we can safely arrive at the conclusion that our strategy significantly improves the performance of the standard t-SNE while the complexity almost remains the same.

## 5. Conclusions

We have developed a preprocessing t-SNE manifold learning algorithm whose preprocessing strategy employs the idea of LE to aggregate each data cluster and the KNN to enlarge the gaps between different clusters. Our approach significantly improves the capability of dimensionality reduction and high-dimensional data visualization of the standard t-SNE while the computation complexity remains almost the same.

We also want to point out that the parameters used in Algorithm 1 (such as $k_{0}$, $k_{1}$, α and $N_{0}$) are determined based on experience. Obviously, different parameters lead to different dimensionality reduction performances and also different complexities. More precisely, the larger the $k_{0}$ or $k_{1}$ the more complex the algorithm; on the other hand, if $k_{0}$ and $k_{1}$ are too small, the structure of the low-dimensional data may be distorted. Thus, it would be interesting to study how to set those parameters to an optimal value to balance performance and complexity. We will consider this as a part of our next work.

## Figures and Tables

**Table 1:** Average running time of SNE, t−SNE and our preprocessing approach on the visualization of 5000 data randomly selected from MNIST. The simulation platform is a laptop with Intel CPU i7−6700HQ.

Algorithm	SNE	t-SNE	Our Preprocessing Strategy
Running Time	530.09 s	278.62 s	283.71 s

## Data Availability

No new data were created.
